# Sequence variations of phase-separating proteins and resources for studying biomolecular condensates: Resources for studying biomolecular condensates

**DOI:** 10.3724/abbs.2023131

**Published:** 2023-07-18

**Authors:** Gaigai Guo, Xinxin Wang, Yi Zhang, Tingting Li

**Affiliations:** 1 Department of Biomedical Informatics School of Basic Medical Sciences Peking University Health Science Center Beijing 100191 China; 2 Key Laboratory for Neuroscience Ministry of Education/National Health Commission of China Peking University Beijing 100191 China

**Keywords:** phase separation, sequence variation, biomolecular condensate, disease

## Abstract

Phase separation (PS) is an important mechanism underlying the formation of biomolecular condensates. Physiological condensates are associated with numerous biological processes, such as transcription, immunity, signaling, and synaptic transmission. Changes in particular amino acids or segments can disturb the protein’s phase behavior and interactions with other biomolecules in condensates. It is thus presumed that variations in the phase-separating-prone domains can significantly impact the properties and functions of condensates. The dysfunction of condensates contributes to a number of pathological processes. Pharmacological perturbation of these condensates is proposed as a promising way to restore physiological states. In this review, we characterize the variations observed in PS proteins that lead to aberrant biomolecular compartmentalization. We also showcase recent advancements in bioinformatics of membraneless organelles (MLOs), focusing on available databases useful for screening PS proteins and describing endogenous condensates, guiding researchers to seek the underlying pathogenic mechanisms of biomolecular condensates.

## Introduction

Biomolecular condensates organize cellular components in a temporally and spatially appropriate manner. It has been shown that the formation of some MLOs is initiated by liquid-liquid phase separation (LLPS) of particular proteins [ 1– 3] . There are other cases where proteins switch to highly ordered solid-like phases, such as the fibrillar assemblies of huntingtin exon 1 proteins in Huntington’s disease [4]. The condensed state can improve the efficiency of cellular processes, which are involved in a range of cellular processes, including transcription, chromatin organization, RNA processing, protein homeostasis, innate immunity, cell-cell adhesions, signaling, and synaptic transmission [ 5– 12] . For example, an increase in local molecular concentration facilitates the reaction rate of enzymes, such as cyclic GMP-AMP synthase (cGAS), in innate immune signaling [7]. These condensates are usually linked to a subset of phase-separating proteins, which in turn govern their components and material states. Thus, variations observed in such protein-coding sequences can substantially impact the functions of biomolecular condensates, which contribute to various diseases.

Dysfunctional condensation is implicated in a variety of pathological processes linked to liquid- or solid-like condensates. For example, mutations of methyl CpG binding protein 2 (MeCP2) can disrupt heterochromatin assembly, which forms via LLPS in native status and develops into transcriptional dysregulation in Rett syndrome [13]. Gain of LLPS propensity is observed frequently in tumorigenesis, such as in the manner of in-frame fusion with IDRs or LCDs [ 14– 16] . In addition, numerous disorders are caused by liquid-solid transitions [17]. Irreversible mutations of many RNA binding proteins can contribute to degenerative diseases, such as mutations of TDP-43, hnRNPA1, and FUS in amyotrophic lateral sclerosis/frontotemporal dementia (ALS/FTD) [ 9, 18, 19] .

In this review, we first characterize four types of sequence variations associated with dysfunctional condensates. We then provide a collective assessment of current computational resources for describing proteins driving liquid-like PS or solid-like phase transition, as well as their relations with disease-associated condensates.

## Interaction Modes and Theories of Phase Separation

**Figure 1:**
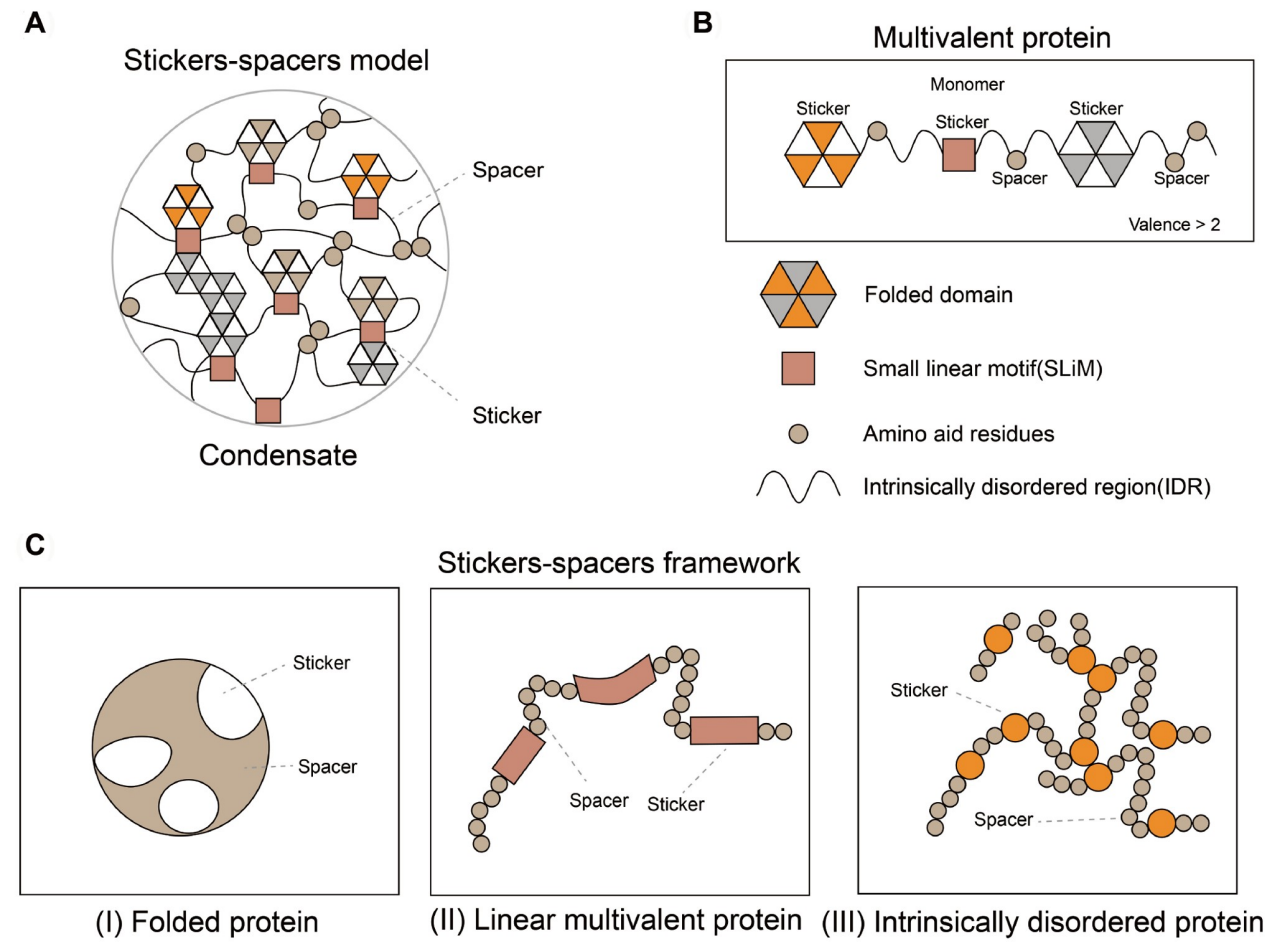
Figure 1 Theories of phase separation (A) Comprehensive composition of membrane-less organelles (MLOs) that consist of phase-separating proteins driven by diverse forces. Biological MLOs are frequently a mixture formed through homotypic and heterotypic attractions between phase-separating proteins. (B) Phase-separating proteins are considered linear polymers that can interact with each other via attractive groups of different types. Polymer proteins are multivalent. Most of these proteins have at least two types of attractive groups, including domains for polymerization, linear motifs, or specific amino acid-rich regions, which are linked by spacers. (C) Three modes of systems with conceptually defined stickers and spacers, including: (I) folded proteins, in which the interaction patches are stickers and the regions on the folded domain are spacers that serve as the surface scaffold for stickers; (II) linear multivalent proteins, in which the folded binding motifs are stickers for molecule recognition. Spacers are linkers connecting those domains; and (III) intrinsically disordered proteins (IDPs), in which stickers are single residues, short linear motifs, or some combination of the two.

Theories that try to explain PS proteins in a universal mechanism often emphasize the importance of multivalency [ 20, 21] . One of the most well-studied theories is the stickers-spacers framework ( Figure 1A) [ 22– 24] . Multivalent proteins are generally constructed of diverse attractive groups, including amino acid residues, modular domains, or emerging stickers that function in self-oligomerization ( Figure 1B). Spacers are interspersed between stickers and are, on most occasions, considered not to affect PS directly. In this theory, stickers are grouped into three conceptual modes in terms of the molecular identities of stickers ( Figure 1C). For folded proteins, the surfaces of folded domains can be considered stickers. For linear multivalent proteins, stickers may appear sporadically along the sequences as regional complexes. For intrinsically disordered regions (IDRs), stickers are likely a particular type of residue (aromatic, charged, or polar amino acids), short linear motifs (SLiMs), or modular domains involved in biomolecular recognition. According to the stickers-spacers theory, we next briefly discuss how sticker valence, interaction strength, and spacer patterning influence the phase behavior of PS proteins.

## Driving Force of Phase Separation

### Types of protein stickers

Stickers can be distinguished by employing a series of computational [ 25, 26] , theoretical [27], and experimental approaches [ 28– 30] , most of which are based on the well-known architectures of multivalent proteins or those binding with RNA molecules [ 29, 31, 32] . Conceptually, these biological stickers can be categorized into three classes: folded domains, specific linear motifs, and individual residues in IDRs [ 22, 27] . The multivalent condensates can originate from cation-π, π-π, charged-charged, or dipole-dipole interactions ( Table 1). Alternatively, emergent stickers can also drive condensate formation through homotypic attractions, namely, oligomerization or polymerization, such as the coiled-coil (CC) domain of ORF1 [34] and the sterile alpha motif (SAM) of Shank3 [35].

**Table:** **Table 1** Representative phase-separating proteins related to important biomolecular condensates or diseases

Protein	Material property	Condensate	Attractive sticker	Biological processes	Ref.
Residue-rich IDR-mediated condensate				
NPM1	Liquid	Nucleolus	S/R-rich	Anaplastic large cell lymphoma (NPM1-ALK fusion protein)	[36]
HOXD13	Liquid	Transcriptional condensate	A-rich	Hereditary synpolydactyly (expansion of polyalanine)	[37]
G3BP1	Liquid, solid	Stress granule	E-rich	Activates or modulates innate and adaptive immune responses	[ 31, 38]
RBGD2/4	Liquid	Stress granule	F/Y-rich	Heat resistance regulation	[39]
FXR1	Liquid	mRNA transportation and translation related condensate	S/R-rich	Congenital multi-minicore myopathy (alternative splicing)	[40]
SRSF2	Liquid	Nuclear speckle	S/R-rich	RNA splicing and spliceosome assembly	[41]
UBQLN2	Solid	Stress granule	P-rich	Regulation of autophagosome assembly; ubiquitin-dependent protein catabolic process	[ 42, 43]
hnRNPDL	Liquid, solid	mRNA transportation and translation related condensate	R/Y-rich, G/T-rich	Transcription repression	[44]
FUS	Solid	Stress granule	G/Y/R-rich, polyS/G	Myxoid liposarcoma (FUS-CHOP); ALS/FTD (missense mutation)	[ 29, 45]
SAFB	Liquid	Transcriptional condensate	R/G-rich	Pericentromeric heterochromatin organization	[46]
ARID1A	Liquid	Spliceosome	P/S-rich	Recognition of large constitutive exons	[47]
K-Ras4B	Liquid	P granule	K-rich	GTPase, regulating proliferation, apoptosis and cell growth	[48]
U1-70K	Liquid	Nuclear body	D/E/R/K-rich	RNA splicing	[49]
FBL	Liquid	Nucleolus	G/A-rich	Ribosomal RNA processing	[50]
Prospero	Liquid	Heterochromatin	Aromatic/nonpolar residues	Regulation of terminal neuronal differentiation	[51]
Cavin1	Liquid, hydrogel	Caveolae-related condensate	D/E -rich	Caveolae formation and organization	[52]
CYCT1	Liquid	Nuclear speckle	H-rich	Regulation of cell cycle	[53]
FLOE1	Liquid	Desiccation-related condensate	Q/P/S-rich	Response to desiccation stress	[54]
TDP-43	Liquid, solid	Neuronal granule	Q/N-rich	RNA biogenesis and processing	[ 55, 56]
Motif/domain-mediated condensate				
GRB2-SOS	Hydrogel	Receptor cluster	PRM_SH3	Signal transduction (downstream of RTK signaling pathway)	[57]
Nephrin-NCK	Liquid	Receptor cluster	pT_SH2	Signal transduction (downstream of RTK signaling pathway)	[58]
PML	Liquid	PML nuclear body	SIM_SUMO	Regulation of apoptosis and growth suppression	[1]
MATR3	Liquid	RNA-binding condensate	RRM_RNA	Innate immune response	[59]
cGAS	Liquid, solid	DNA-binding condensate	DNA binding domain	Innate immune response	[7]
TRF1/2	Liquid	DNA-binding condensate	DNA binding domain	Telomere maintenance	[60]
Edc3-Pdc1	Liquid	P-body	LSm_HLM	Deadenylation-independent decapping of nuclear-transcribed mRNA	[61]
SPOP/DAXX	Liquid, solid	Nuclear speckle	BTB_BACK	Prostate cancer (missense mutation)	[8]
Nucleocapsid	Liquid	Stress granule	Zinc-finger	HIV-1 viral nucleocapsid	[62]
RAD23	Liquid	Proteasome containing foci	UBL_UBA	Protein degradation	[63]
SynGAP-PSD95	Liquid	Postsynaptic density	PBM_PDZ	Signal transduction (downstream of glutamate receptor)	[12]
Pon	Liquid	Cell polarization-related condensate	FxNxx[F/L]NP[F/Y]E[V/I]xR	Asymmetric cell division	[64]
TNRC6B-Ago2	Liquid, solid	miRISC	G/W_PIWI	RNA-mediated gene silencing	[65]
Repeats-mediated condensate				
LAF-1	Liquid	P granule	RGG	ATP-dependent RNA helicase	[66]
NUP98	Solid	Nuclear pore complex	GLFG	Acute myeloid leukemia (NUP98 fusions with HOXA9, KDM5A, or LNP1)	[67]
PGL-3	Liquid	P granule	RGG	Germline development regulation	[68]
EMB1579	Liquid	Transcriptional condensate	RE/RD/RED	Regulation of DNA-templated transcription	[69]
C9orf72	Liquid	Guanine nucleotide exchange-related condensate	PR/GR/GA	ALS/FTD (expansion of polyPR/GR/GA)	[70]
RNA Pol II	Liquid	Transcriptional condensate	YSPTSPS	DNA-dependent RNA polymerase	[71]
MDC1	Liquid	DNA repairing foci	PST	DNA double-strand breaks repairing factors	[72]
tau	Liquid, solid	Microtubule-related condensate	pseudo-repeat region (~31 aa)	Promotion of microtubule assembly and stability	[73]
Polymerization domain-mediated condensate			
Shank3	Liquid	Postsynaptic density	SAM domain	Signal transduction (downstream of glutamate receptor)	[35]
EML4	Solid	Actin-binding condensate	Coiled-coil	Non-small cell lung cancer (ELM4-ALK)	[ 14, 74]
Huntingtin	Liquid	Huntingtin inclusion	polyQ (β-sheet)	Huntington’s disease (expansion of polyglutamine tract)	[ 75, 76]
hnRNPA1	Liquid, solid	Stress granule	G-rich (β-sheet)	Multisystem proteinopathy; ALS/FTD (missense mutation)	[9]
PrP	Solid	Aβ oligomers	Polybasic PLD	Prion diseases	[77]
AfrLEA6	Liquid, solid	Stress granule	SMP domain	Desiccation tolerance	[78]
TFEB	Liquid	Autophagy-related condensate	bHLH region	Regulation of autophagy and lysosomal biogenesis	[79]
Osh4	Solid	Stress granule	OSBP-related domain	Lipid transportation and direction of cell polarized growth	[80]
53BP1	Liquid	Heterochromatin	Oligomerization domain	Regulation of DNA double strand break repairment	[81]
Dvl-2	Liquid, solid	Wnt pathway-related condensate	DIX domain	Upstream Wnt signaling effector	[ 82, 83]
EBNA2	Liquid	Chromatin	Dimerization domains	Activation of the host resting B-cell and stimulation of B-cell proliferation	[84]
TMF	Liquid	Transcriptional condensate	Oligomerization domain	Recognition of ROS and regulation of flowering transition	[85]

^a^Abbreviations: ALS/FTD, amyotrophic lateral sclerosis/frontotemporal dementia; PML, promyelocytic leukemia; RTK, receptor of tyrosine kinase; SH2, src homology 2; SIM, SUMO-interacting motif; SUMO, sentrin/small ubiquitin-like modifier; RRM, RNA recognition motif; HLM, helical leucine-rich motif; BTB, Broad-complex, Tramtrack and Bric-à-brac; BACK, BTB and C-terminal Kelch; UBL, ubiquitin-like; UBA, ubiquitin associated; PBM, PDZ binding motif; PDZ, post-synaptic density-95, disks-large and zonula occludens-1; PIWI, P-element-induced wimpy testis; SAM, sterile alpha motif; SMP, synaptotagmin-like mitochondrial-lipid binding protein; bHLH, basic helix-loop-helix; OSBP, oxysterol-binding protein; ROS, reactive oxygen species.

^b^Driving factors, effects, and related diseases of phase-separating proteins are listed.

^c^The categories and positions of driving forces are distinguished according to the original literature.

### Parameters of effective stickers

The driving force for PS is governed by three factors, including the effective valence of stickers, the interaction strengths between stickers, and the patterning of spacers in the protein sequence [ 22, 27] . The multivalence of interaction domains or motifs represents a hallmark of proteins that drive phase transitions [20], such as the heterotypic interactions mediated by poly-SIM/poly-SUMO segments [86] and PRM-binding SH3 domains (GRB2-SOS) [57]. Increasing the sticker valence or interaction strength can lower the saturation concentration (c _sat_) and thus expand the PS propensity, similar to the effects of modulating the aromatic residues in hnRNPA1-LCD [87]. Additionally, the reduction of valency via destruction of self-oligomerization can also influence phase behavior, such as the CC domain in ORF1 [34] and SAM domains in the Polycomb Repressive Complex 1 (PRC1) subunit Polyhomeotic (Ph) [88]. For example, Newton and coworkers [34] demonstrated a cooperative interaction between the LCD and CC domain of ORF1, of which both mutations of essential residues in the LCD or CC domain can lead to a repressed PS.

It has been reported that amino acids with similar chemical properties can demonstrate various sticker strengths. Bremer *et al*. [89] found that Tyr is stronger than Phe as a sticker, while Lys weakens the sticker-sticker interaction, such as in the case of prion-like domain (PLD) proteins. Additionally, a few other studies have discovered that clustering and segregation of stickers along linear sequences can regulate the biological functions of corresponding proteins by affecting the driving force of PS [ 90, 91] . A prediction-based study from Das *et al*. [91] showed that the strength of sticker-sticker interactions will increase when charged stickers are segregated along the PLD regions. Additionally, it was recently demonstrated that charged blocks are required for specific compartmentalization and that the IDRs of similar patterned charged blocks present similar and selective functions [92]. However, the segregation pattern of aromatic residues can lead to pathological and irreversible aggregation, such as the PLD of hnRNPA1 [87]. In contrast, uniform distribution of aromatic residues (Tyr) will promote LLPS, such as two RNA binding proteins, RBGD2 and RBGD4 [39].

### Impacts of spacers

Spacers affect the phase behaviors and material properties of condensates by influencing the effective solvation volume (v _es_) of PS proteins [22]. The stickers-and-spacers framework can be well applied to PLD proteins. A decrease in v _es_ stabilizes the PS process of A1-LCD, a homolog of hnRNPA1, when the Ser content is increased [89]. It is also possible to increase v _es_ by increasing the charged content [93]. Under specific circumstances, spacers behave as attractive groups and provide multivalent cross-links [94]. Spacers are typically composed of Ser, Gly, and Gln residues, while poly-residue tracts perform their duty as spacers in an alternative way. Kar *et al*. [29] reported that peptides with uniformly distributed Gly do not form fibrils or gels but can promote self-assembly in the form of poly-Gly tract. Some poly tracts can lead to pathological consequences, e.g., phase transitions of some poly-Gln proteins could initiate irreversible pathological aggregation. Peskett and coworkers [4] found that Huntingtin (Htt) exon 1, a Gln- and Pro-rich region, drives the Htt protein to form solid-like assemblies.

## Sequence Variations and Regulation of Phase Separation

**Figure 2:**
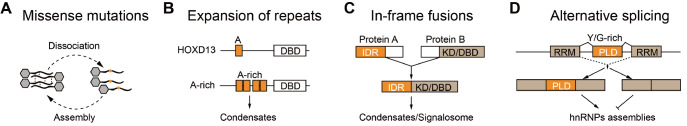
Figure 2 Schematic of four types of gene variations (A) Missense mutations. The impact of missense mutations on phase-separating proteins is two-sided, which is, to some extent, dependent on the structure and general properties of the sequence where mutations occur. (B) Expansion of repeats. An instantiation related to the expansion of Ala-rich repeats. Synpolydactyly associated repeat expansions enhance phase separation of HOXD13-IDR. IDR, intrinsically disordered domain; DBD, DNA binding domain; RRM, RNA recognition motif; PLD, prion-like region. (C) Gene fusions. Functional domains (KD/DBD) fused with phase-separation-prone domains (IDR) can enhance their activities, such as constituent activation of the receptor tyrosine kinase (RTK) pathways in the cellular signalosome. KD, kinase domain; DBD, DNA binding domain. (D) Alternative splicing. The inclusion of exons harboring Y/G-rich PLD promotes multivalent assemblies. For example, the alternative splicing of hnRNPs contributes to the control of global splicing and signaling regulation.

Dysregulation of phase transitions of biomolecule condensates is linked to a series of pathogenic conditions, including the toxicity induced by the liquid‒solid transition of ALS/FTD mutations in FUS (R522G, R524S, P525L, and R495X) [19] and the promotion of tumorigenesis for gain of PS properties, such as NUP98 fusion oncoproteins (NUP98-HOXA9 and NUP98-KDM5A) in leukemic transformation [95]. In particular, many natural mutations in IDRs are associated with diseases [96]. To systematically understand the relationship between sequence variations and condensate dysfunction, we summarize the reports on three type of disease-associated variations (missense mutations, expansions of peptide repeats, and gene fusions) involved in aberrant condensates and one type of post-transcriptional modification process, alternative splicing (AS) ( Figure 2). Based on current findings, we attempt to recapitulate the underlying mechanisms that may be implicated in the pathogenic behaviors of dysregulated condensates.

### Missense mutation

Missense mutations are a critical type of variation observed in proteins that affect biomolecular assembly. To obtain the overall landscape of functional mutations of PS proteins, we referred to PhaSepDB, which provides a collection of manually curated PS-related proteins [97]. Missense mutations altering the phase behaviors of condensates often occur in diverse conformational regions, such as IDRs, modular domains, or polymerization domains ( Figure 2A). Regardless of the complexity and structure of regions harboring mutations, the impacts of mutations on a protein’s phase behavior can be two-sided. For example, mutations of aromatic to hydrophobic residues (F291S and Y283S) observed in the IDRs of hnRNPA2 can slightly repress its homotypic assembly, while mutations near the sites D290V and P298L will enhance its condensed status [98]. Interestingly, a number of mutations have been discovered in domains that function in protein polymerization, such as the monomeric SPOP mutant SPOP ^mutBTB/BACK^. The mutant tends to diffuse in the nucleus and is unable to localize to SPOP-DAXX bodies in HeLa cells [8] Some mutations affect protein phase behavior by monitoring the valency of condensates. Stortz and coworkers [99] found that GR P481R, a constitutively tetrameric glucocorticoid receptor (GR) mutant, presents a higher capability to form condensates, while most monomeric receptor GR mutants (A465T/I634A) do not form foci in cells. In summary, the perturbation of protein homotypic or heterotypic polymerization is an important manner that affects proteins’ capability to form condensates [ 8, 98, 99] .

In addition to the disorder status and frequency of particular mutations, the physiochemical properties of drivers are relatively preserved in the regulation of PS. Previous study shows that mutating attractive groups into hydrophilic amino acids partially accounts for the loss of PS propensity. For example, the W131G mutation is expected to have an impact on SPOP phase behavior by increasing its v _es_, according to the stickers-spacers model [8]. Conversion to hydrophobic or charged amino acids can accelerate the transition from the liquid state to the solid-state or fibrils. These interacting stickers include aromatic, acidic, or basic amino acids, which are observed frequently in IDRs or low complexity domains (LCDs), such as the FUS G156E mutant, hnRNPA1 D262V mutant, and hnRNPA2 R521C mutant [ 32, 98] . Changes in charged residues often affect the phase behaviors of many posttranslationally modified proteins, such as methylated hnRNPA2 [100] and phosphorylated NPM1 [92]. Nevertheless, the regulation of PS is complicated and context-dependent in cells. These findings demonstrate that appropriate sequence modifications are advisable in *de novo* design for manipulating PS.

### Expansion of pathogenic repeats

Expansion of sequence repeats is another key type of disease-associated variation that occurs in dozens of inherited human disorders [ 101, 102] . Previous reports indicated that a majority of sequence expansions are observed in transcription factors (TFs) and proteins associated with neurodegenerative diseases [103]. Expansions of repeated sequences alter their phase behaviors and abilities to recruit regulators, leading to pathogenetic assembly and aggregation. Basu *et al*. [37] found that the expansion of Ala repeats facilitates the homopolymerization and PS of TF HOXD13 ( Figure 2B), which in turn excludes the incorporation of MED1, a subunit of the Mediator coactivator. Furthermore, changes in components in the HOXD13-containing assembly due to the global expansion of Ala repeats alter the transcriptional program in a mouse model of synpolydactyly. These findings suggest a framework of how the expanding repeats in IDRs of TFs (HOXA13, RUNX2, and TBP) contribute to human pat hologies [37]. Another similar example is the expansion of the GGGGCC repeat in chromosome 9 open reading frame 72 (C9orf72), a key cause of ALS/FTD [104]. The products containing polyGly-Arg (GRn) and polyPro-Arg (PRn) are able to undergo LLPS [70] as toxic dipeptides contribute to pathology. Expanding repeats interact with many other proteins and alter their PS propensity. GRn and PRn repeating sequences perturb the composition of nucleoli, stress granules, and RNA granules, which might play a dominant role in the pathogenesis of ALS/FTD [105]. A well-studied example is the Huntington’s disease-associated protein Htt, which contains a disordered Gln-rich region (PolyQ) in its first exon [106]. Mutant Htt protein (mHtt) harboring PolyQ expansion diffuses slowly and forms more stable and solid aggregates than WT Htt [107]. This example indicated that LLPS might mediate solid-like assembly formation [108]. Li *et al*. [109] discovered that the aggregation of mHtt can reduce its targeting sites and impair gene expression programs in neuronal cells.

### Gene fusions

Fusion genes are a crucial category of cancer drivers that are identified in approximately 20% of cancer morbidity [110]. Among these, in-frame fusions are considered the major events in some cancers, such as BCR-ABL1 in acute myeloid leukemia and EWS-FLI1 in Ewing sarcoma [ 16, 111] . As numerous IDRs or phase-separation-prone regions with the features mentioned above are supposed to be the drivers of PS, it is convincing that fusion proteins with such domains may have a higher potential to undergo PS. In fact, a significant proportion of oncoproteins include such disordered domains, such as PLDs or LCDs. Fusion TFs are an important category of oncoproteins that induce aberrant gene expression [112]. For example, in FET fusion oncoproteins, the disordered PLD is fused with the DNA-binding domains (DBDs) of TFs (CHOP, FLI1, and DDIT3), making PLDs behave as transactivation domains [ 113, 114] ( Figure 2C). These aberrant PLD-DBD fusions condense and recruit the transcriptional machinery and regulators, including RNA polymerase II, BRD4, and mSWI/SNF, which activate transcription independent of upstream stimuli [ 113, 115, 116] . In addition, another group of oncoproteins is NUP98 fusion proteins that share the FG-repeated IDR of NUP98, a component of the nuclear pore complex (NPC) [ 15, 117] . For example, NUP98-HOXA9 promotes the global expression of leukemogenic genes due to an increase in its LLPS ability [95]. In addition to TFs, receptors of tyrosine kinases (RTKs) are another type of cancer driver [118]. Fusion with LCD from a phase-separating protein mediates the kinase domain (KD) function in cytoplasmic protein granules, constitutively activating the RAS pathway without ligand stimuli [74]. Previous evidence has demonstrated a similar mechanism in ALK fusions, such as ELM4-ALK and NPM1-ALK [ 14, 119, 120] .

### Alternative splicing

The length and amino acid composition of attractive groups in PS proteins can also be modulated pretranslationally through AS, which produces distinct mature mRNAs from a single pre-mRNA by including or skipping different exon segments. Approximately 95% of multiexon genes are expected to undergo AS [ 121– 124] , most of which are prone to expression in a tissue-specific manner, such as FXR1 splicing in muscle development [48)] The tissue-specific alternatively spliced exons are such regions where interactions frequently occur [ 125– 127] . AS events are observed as a critical posttranscriptional mechanism that functions on a global scale to rewire cellular responses [ 128– 130] . Interestingly, many studies have revealed that alternatively spliced exons are enriched in IDRs, presumably to maintain functional and regulatory diversity while avoiding the disruption of core protein structure [ 131– 133] . Additionally, IDRs often contain linear motifs that mediate ligand recognition, which regulates the functions of such alternatively spliced IDPs [ 131, 134] .

AS of Ser- and Arg-rich IDRs can achieve different morphologies of condensates, *e.g.*, the splicing of FXR1 in the development of Xenopus. Smith *et al*. [40] reported that cells expressing a longer isoform of FXR1 generate larger and more puncta than those expressing the shorter isoform, which lacks exons 15 and 16. Both isoforms bind with RNAs and present concentration-dependent assemblies in cells. Moreover, they also observed that phosphorylation of the Ser residues in its IDRs is critical to regulating the function of FXR1, as phosphorylation by CK2 reduces the aggregation of the FXR1 isoform with the longer IDR. Thus, both the length of IDRs and the potential of PTMs can regulate the behaviors and functions of AS IDPs in a lineage-specific manner. Similarly, it has been suggested that Ser/Arg-rich splicing factors are likely to be subject to PTMs to modulate disorder status [ 135– 137] .

Previous studies discovered that mammalian exons with specific AS events are enriched in IDRs containing Gly- and Tyr-rich motifs, such as hnRNP families and other RBPs, which play diverse roles in RNA processing [138]. Such AS events are evolutionally conserved in the control of splicing sites. Aberrant assembly of these proteins can lead to the formation of pathological aggregates implicated in many degenerative diseases [139]. Additionally, GY-rich IDRs have been proven to function in the formation of higher-order protein assemblies, including RNA granules, hydrogel-like MLOs, and fibrillar-like structures [ 138, 140, 141] . Skipping or including different exons regulates the phase behavior of hnRNP assemblies ( Figure 2D), which in turn controls global AS events in cells and tissues.

It has been observed that AS regulates the assembly properties of RNA-processing proteins by manipulating the incorporation of IDRs in different isoforms [142]. Batlle *et al*. [44] demonstrated that hnRNPDL isoforms require interactions between Tyr and Arg residues in PLDs to phase separate, which is regulated by AS events. In fact, AS events are frequently observed in PLDs [138] and interfere with the PS property and ability to develop multivalent interactions in proteins, such as in the cases of hnRNP A1 and hnRNP A1B. Compared to the control hnRNP A1 isoform, hnRNP A1B harboring an elongated PLD domain has a greater fibrillization propensity, which is toxic to ALS patients [143].

## Resources for Component Screening

Failure in the regulation of material states may lead to aberrant protein assemblies, which could trigger a range of pathological processes, with the sequence variations mentioned above as a possible prominent mechanism [ 144– 147] . To aid the investigation of aberrant condensate-associated diseases, we provide emerging available databases and prediction methods, which are necessary to describe experimental phenomena, focusing on the survey for potential interactions with PS proteins and the regulatory mechanisms underlying the formation of these aberrant condensates.

### Databases of proteins and RNAs involved in biological condensates

Several LLPS-associated protein and RNA databases were released until 2023. Since the original publications and web interfaces of these resources have already provided detailed explanations on their usage, we aim to provide a guide for users on the strengths of these resources and explain particular usage where they could be beneficial. The main features of the databases discussed are summarized in Tables 2 and 3. We collected features that highlight the basic principles and characteristic differences.

**Table:** **Table 2** Resources for phase-separating proteins and condensates

Name	Lastupdated	Scope	Annotation	Server	Ref.
DrLLPS	January2019	150 scaffold proteins and 987 regulators; relations to 40 biomolecular condensates.	(a) Two categories: Scaffolds (drivers of LLPS) and regulators (regulation of MLOs formation: P bodies or stress granule); (b) basic annotations (protein sequence, modification, *etc.*); (c) genetic variations (SNP, cancer mutations, or gene fusions); (d) molecular interactions (proteins, nucleic acid, or relations to drugs).	llps.biocuckoo.cn	[148]
LLPSDB	July2019	273 PS proteins: 198 natural proteins and 75 designed proteins.	(a) Biomolecular information (protein sequence, modification, *etc.*); (b) experimental conditions (validated in vitro) and phase behavior description; (c) linkage to other open comprehensive databases (Uniprot, MobiDB, or DisProt).	bio-comp.org.cn/llpsdb/	[149]
PhaSePro	September2019	121 PS proteins: 109 eukaryotic, 5 bacterial and 7 viral proteins.	(a) Proteins/regions: LLPS alone or parts of LLPS systems with co-drivers; (b) experiment type and conditions: *in vivo* or *in vitro*; (c) regulation partnersand connections to MLOs and diseases (SNP).	phasepro.elte.hu	[150]
MloDisDB	April2020	73 biomolecular condensates; 719 relationships between MLOs and diseases; 52 relationships between LLPS proteins and diseases; diseases: cancer, nervous system, infectious diseases/anemia, and apoptosis/aging.	(a) Basic information (MLO, factor, and disease information); (b) changes of the MLO (relations, organisms, and cell lines); (c) changes of factors (relation to the MLO-diseases or LLPS-diseases); (d) factors include proteins, RNA, chemicals, oligonucleotides, or peptides.	mlodis.phasep.pro/	[3]
PhaSepDB	June2022	860 PS proteins: 300 PS-self proteins and 560 PS-other proteins; 590 MLO-related proteins (low-throughput) and 5292 MLO-related proteins (high-throughput).	(a) Two categories: PS-self proteins (PS by themselves *in vitro*) and PS-other proteins (PS with partners *in vitro*); (b) PS experiments: droplet state, phase diagram, *in vivo* or *in vitro*; (c) PS regulating macromolecules:co-PS protein, RNA/DNA, and chemicals; (d) PS regulating events on proteins: PTM, mutation, oligomerization, repeat region and alternative splicing.	db.phasep.pro/	[151]

SNP, single nucleotide polymorphism; LLPS, liquid-liquid phase separation; PTM, post-translational modification; MLO, membrane-less organelle.

**Table:** **Table 3** Resources for RNAs related to phase separation and condensates

Name	Lastupdated	Scope	Annotation	Server	Ref.
RNAPhaSep	June2021	325 RNAs: validated 22 organisms.	(a) Basic information: ID, sequence, function, modification, and location; (b) binding partner: RNAs or proteins.	rnaphasep.cn	[152]
RPS	June2021	337 RNAs: validated; 20,153 RNAs: high-throughput analyses; 1358 RNAs: predicted.	(a) RNAs type: protein-coding sequence, lncRNA, pseudogene, or virus RNA; (b) putative sequence features: motif, modification, 2D/3D structures, or binding site with partners; (c) relations to diseases; (d) condensates type: nucleus, cytoplasm, and others.	rps.renlab.org	[153]

DrLLPS is an integrative database for the proteins that can undergo LLPS [148]. It classified PS proteins into two categories, *i.e.*, scaffolds and regulators, which were assigned to 40 biomolecular condensates. This database also documents PS-associated proteins in 164 eukaryotes through computational prediction. LLPSDB is a web-accessible database providing curated LLPS proteins and corresponding *in vitro* experimental conditions from published literature [149]. It provides the details for 273 PS proteins associated with 1182 experiments. LLPSDB annotated proteins with biomolecular data, such as protein sequence or protein modification, and specific PS information, such as experimental conditions and phase behavior descriptions. It is useful for the study of the biophysical background of LLPS. PhaSePro is a manually curated database that provides information on LLPS proteins with structural data. It describes proteins that can drive PS alone or as part of well-defined multicomponent systems [150]. While the database has only 121 proteins, it provides reliable PS drivers. MloDisDB is a curated collection of the relations between MLOs and diseases and the relations between LLPS and diseases [3]. It contains up to 771 relations with the corresponding diseases from 607 publications, which might facilitate the investigation of the pathogenic mechanism underlying PS-related diseases. The LLPS proteins in MloDisDB are the collection of the reviewed proteins from the DrLLPS, LLPSDB, PhaSePro, and PhaSepDB databases. PhaSepDB is a centralized resource that provides experimentally verified PS proteins and MLO-related proteins [97]. The database currently contains 300 PS-self proteins and 560 PS-other proteins. It provides detailed annotations for PS proteins, including the material states of PS droplets, regions used in experiments, phase diagrams, PS partners (protein, RNA, and others), and regulatory factors of PS ability (mutation or modification). The PS proteins in PhaSepDB are annotated with verification experiments based on original publications, such as droplet formation and fluorescence recovery after photobleaching (FRAP) experiments performed *in vivo* or *in vitro*. PhaSepDB is a suitable resource for those who want to gain an overview of *in vitro* and *in vivo* experiments performed in proof of LLPS of a target protein.

Increasing evidence has demonstrated the essential roles of RNAs in the functions and maintenance of MLOs such as nucleoli, P-bodies, and stress granules. At present, two resources have addressed and collected RNAs related to PS and condensates ( Table 3). RNAPhaSep is a curated database that contains self-assembling RNAs or RNAs and proteins, which can undergo cophase separation in validated experiments [152]. RPS integrates 21,613 LLPS-related RNAs with validated, high-throughput, and predicted evidence [153]. It provides RNAs with informative annotations, such as putative motifs, structures, modification sites, or related diseases. Both databases serve as valuable platforms for the investigation of RNA-related PS and biological processes.

### Prediction tools for phase-separating proteins

A range of computational algorithms have been developed to predict PS proteins, which are to some extent applicable for proteins with higher contents of IDRs or LCDs. PScore predicts long-range π-π interactions based on statistically expected interactions between π-orbital-containing residues [154]. π-π interactions can occur between residues containing aromatic rings (Tyr, Phe, Trp, and His) as well as amino acids with π-side chains (Gln, Asp, Glu, and Arg). PLAAC predicts disordered PLDs using a hidden Markov model (HMM) [155]. PLAAC was trained on the yeast proteome and extended to screen human proteins [156]. ZipperDB collects protein regions predicted to form fibrils using segments from more than 20,000 putative amyloid-forming sequences. It supports the analysis of individual prediction from user input [157]. catGRANULE is a more generic prediction algorithm and excels at predicting dosage-sensitive proteins. It predicts the tendency of proteins to localize in cytoplasmic foci by combining nucleic acid binding propensities, structural disorder, sequence length, and amino acid composition [158]. The PSPer approach predicts and screens proteins with similar characteristics to the FUS-like phase-separating regions, including PLDs, RNA binding motifs, and disordered arginine-rich regions [159]. FuzDrop is a method to predict droplet-promoting regions and proteins, which can spontaneously undergo LLPS, providing a sequence-based profile of propensities [160].

Most current predictors only perform well in proteins that can self-assemble to undergo PS (SaPS). PhaSePred is a machine-learning predictor that can predict the potential of proteins to assemble through partners, considered partner-dependent PS (PdPS) proteins [151] and SaPS proteins. By incorporating multimodal features such as phosphorylation level and immunofluorescence (IF) images, PhaSePred can discriminate proteins located in human MLOs, such as the components recorded in the OpenCell nuclear puncta set, the DACT1-particulate proteome set, the G3BP1 proximity labelling set, and the PhaSepDB high-throughput set. It provides a web server that integrates other well-used PS predictors, such as PLAAC, Pscore, and catGRANULE.

The discovery and prediction of protein constituents in biological condensates present enormous opportunities for uncovering underlying mechanisms. The DeepPhase method provides a global view of the human PS proteome, according to the morphology of their condensates, instead of using sequence-dependent traits such as other predicting tools [161]. Based on the shape of targeted proteins in IF images from the Human Protein Atlas (HPA), DeepPhase scores the PS proteins in a cell-type specific manner, which can inform users with appropriate cell line types, as well as antibody choices for the proteins they are interested in. DeepPhase also suggests that a subset of kinases and TFs with higher predicting scores tend to display significantly reduced substrate specificity.

Informative reviews have comprehensively summarized the route performance and primary principle, which suggests a limited overlap between existing predictors that may generate a significant rate of false negatives [ 162– 164] . The molecular mechanism underlying PS is complicated and involves diverse molecular components with distinct roles, *e.g.*, partner-dependent PS proteins and regulators. Prediction efficiency depends on standard negative and positive phase separating protein datasets, which are thus far limited. Indeed, these predictors successfully predict numerous phase-separating proteins, confirming the importance of phase-separation-prone features and providing the foundation to establish more integrative and accurate predictors or tools for protein science in the future [ 26, 165] .

## Conclusions and Perspectives

It is clear that PS is presumed to play ubiquitous roles in fundamental biological processes. The question is thus raised as to whether PS is a requisite in a given function. A detailed experimental pipeline for LLPS-associated studies has been provided by Gao and coworkers, addressing how we can relate the experimental phenomena to biological functions [166]. The morphological and topological characteristics of target proteins are dependent on image information. Therefore, the robustness is limited to optical resolution. In addition, PS can be driven and regulated by protein-protein, protein-ion, and protein-nucleic acid interactions, which suggests the essentiality of PS-related networks when judging the roles of target proteins in biological processes.

Increasing numbers of useful bioinformatics methods have been developed to predict PS proteins. However, most of these methods are designed for screening such proteins with higher IDR content [163], such as PScore for π-π enriched segments [154], PLAAC for PLDs [155], and FuzDrop for droplet-promoting regions and proteins [160]. Proteins with ordered structures or modular domains account for a large subset of PS proteins as well, such as HP1A [167], UTX [168], and DVL2 [ 82, 83] . The computational tools to identify such proteins with lower IDR content are insufficient. Nevertheless, it may not be so challenging to identify such proteins. For example, the DeepPhase method can distinguish and predict some condensed proteins enriched in the WD40 motif [161], which is considered a feature of the PS protein Gro [169]. The PhaSePred method can also predict a set of proteins with structured domains, such as SH3 and PDZ domains [151]. Thus, further developments in computational methods should consider more comprehensive features in addition to sequence composition.

A range of diseases are associated with gene mutations, which can lead to the gain or loss of the ability to condense during the development of pathogenesis [ 6, 8, 147, 170] . For example, the typical prostate cancer mutations in SPOP, W131G and F133V, disrupt SPOP and DAXX colocalization, resulting in decreased substrate ubiquitination [8]. However, under many other pathologic conditions, condensation of particular proteins underlies disease progression, such as KEAP1 (R320Q) in lung cancer [6] and PTPN (Y279C) in liver cancer [170]. It is promising to model the precise effects of sequence mutations on the phase behavior of proteins, especially those related to diseases. Therefore, future bioinformatics approaches should provide more insights into the effects of sequence alterations, such as missense mutations, aiding in providing a mechanistic understanding of disease mutations. Therapeutic approaches targeting such aberrant condensates might restore their physiological states, as in the case of myricetin inhibiting droplet formation of the tau protein [171], inspiring further interest in the therapeutic area.
